# Characterization of whole-genome autosomal differences of DNA methylation between men and women

**DOI:** 10.1186/s13072-015-0035-3

**Published:** 2015-10-19

**Authors:** Paula Singmann, Doron Shem-Tov, Simone Wahl, Harald Grallert, Giovanni Fiorito, So-Youn Shin, Katharina Schramm, Petra Wolf, Sonja Kunze, Yael Baran, Simonetta Guarrera, Paolo Vineis, Vittorio Krogh, Salvatore Panico, Rosario Tumino, Anja Kretschmer, Christian Gieger, Annette Peters, Holger Prokisch, Caroline L. Relton, Giuseppe Matullo, Thomas Illig, Melanie Waldenberger, Eran Halperin

**Affiliations:** Research Unit of Molecular Epidemiology, Helmholtz Zentrum Muenchen, German Research Center for Environmental Health, Neuherberg, Germany; Institute of Epidemiologie II, Helmholtz Zentrum Muenchen, German Research Center for Environmental Health, Neuherberg, Germany; Blavatnik School of Computer Science, Tel-Aviv University, Tel-Aviv, Israel; German Center for Diabetes Research (DZD e.V.), Neuherberg, Germany; Human Genetics Foundation-Torino, Turin, Italy; Department of Medical Sciences, University of Torino, Turin, Italy; MRC Integrative Epidemiology Unit, School of Social and Community Medicine, University of Bristol, Bristol, UK; Institute of Genetic Medicine, Newcastle University, Newcastle upon Tyne, UK; Institute of Human Genetics, Helmholtz Zentrum Muenchen, German Research Center for Environmental Health, Neuherberg, Germany; Institute of Human Genetics, Technical University Munich, Munich, Germany; Epidemiology and Public Health, Imperial College London, London, UK; Epidemiology and Prevention Unit, Fondazione IRCSS Istituto Nazionale Tumori, Milan, Italy; Dipartimento di Medicina Clinica e Chirurgia, Federico II University, Naples, Italy; Cancer Registry and Histopathology Unit, “Civile-M.P. Arezzo” Hospital, ASP 7, Ragusa, Italy; Hannover Unified Biobank, Hannover Medical School, Hannover, Germany; Institute for Human Genetics, Hannover Medical School, Hannover, Germany; Department of Molecular Microbiology and Biotechnology, George Wise Faculty of Life Science, Tel-Aviv University, Tel-Aviv, Israel; International Computer Science Institute, Berkeley, CA USA

**Keywords:** DNA methylation, EWAS, Sex, Enrichment analysis, CpG, Imprinting, KORA, ALSPAC, EPICOR

## Abstract

**Background:**

Disease risk and incidence between males and females reveal differences, and sex is an important component of any investigation of the determinants of phenotypes or disease etiology. Further striking differences between men and women are known, for instance, at the metabolic level. The extent to which men and women vary at the level of the epigenome, however, is not well documented. DNA methylation is the best known epigenetic mechanism to date.

**Results:**

In order to shed light on epigenetic differences, we compared autosomal DNA methylation levels between men and women in blood in a large prospective European cohort of 1799 subjects, and replicated our findings in three independent European cohorts. We identified and validated 1184 CpG sites to be differentially methylated between men and women and observed that these CpG sites were distributed across all autosomes. We showed that some of the differentially methylated loci also exhibit differential gene expression between men and women. Finally, we found that the differentially methylated loci are enriched among imprinted genes, and that their genomic location in the genome is concentrated in CpG island shores.

**Conclusion:**

Our epigenome-wide association study indicates that differences between men and women are so substantial that they should be considered in design and analyses of future studies.

**Electronic supplementary material:**

The online version of this article (doi:10.1186/s13072-015-0035-3) contains supplementary material, which is available to authorized users.

## Background

There is no doubt that men and women are different. Differences exist in risk and incidence of diseases between males and females, and sex is an important component of any investigation of the determinants of phenotype or disease etiology [1, 2]. At the molecular level, it has been shown that metabolic profiles of men and women differ substantially [3], whereas genomic differences on the SNP level are not reproducible [4]. However, it remains largely unknown to what extent men and women differ at the epigenomic level. Shedding light on the differences between men and women in terms of DNA methylation (DNAm) is particularly important in conducting epigenome-wide association studies (EWAS). Insights on extent and distribution of sex-dependent DNAm can potentially improve the identification of disease- or phenotype-related methylation signatures.

The relation between DNAm and sex in humans has been studied previously [5–8]. These studies, however, were limited in their scope and in their statistical power. In particular, several earlier studies considered ‘global’ measures of DNAm that captured repeated, noncoding regions in the genome (e.g., LINE-1 and Alu repeats [5, 9–12]). Others have focused on specific pathways or loci (e.g., cancer sites [13], specific genes [11, 14], low numbers of CpGs across the genome [15]), or restricted their analysis to certain chromosomes [16, 17].

In the current study, we aimed to systematically analyze whole blood DNAm differences between sexes across the genome in a large population-based cohort, and to validate our results in three additional cohorts. In addition, we sought to characterize the genomic loci that show substantial DNAm differences between men and women by means of different enrichment analyses and expression analysis.

## Results

### Discovery of sex-methylation associations

**Fig. 1:**
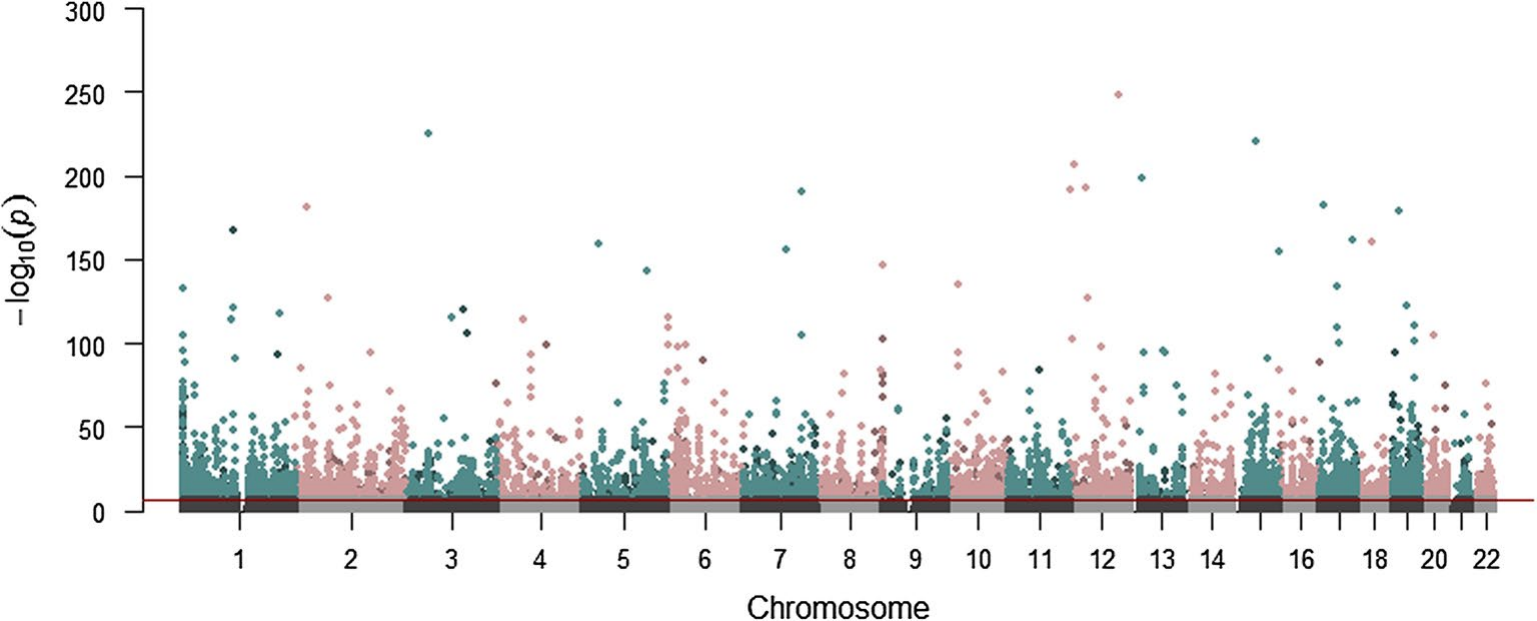
Genome-wide sex differences in DNAm across the autosomes in the discovery study KORA F4 (Manhattan plot). Chromosomes (autosomes) are represented by alternating *colors* with the *lighter* color per chromosome representing hypermethylated CpGs and the *darker* color hypomethylated CpGs (male versus female). The *red line* represents the significance level of 1.26E−07

In our discovery sample from the population-based KORA F4 study, we sought to identify DNAm differences between 877 men and 922 women at 470,920 autosomal CpGs. Details on the cohort are given in Table 1. Association analysis of normalized methylation data (beta-values) from 391,885 CpGs revealed 11,010 sex-methylation associations (SMAs) (*p* < 1.26E−07) with some of them being highly significant (*p* < 1E−300). The sex-associated CpGs were spread across the autosomal genome (Fig. 1). These and all subsequent significance levels were corrected for multiple testing according to the Bonferroni method. Additional file 1 provides a flow-chart of our analyses.

**Table 1:** Population characteristics of discovery sample KORA F4, replication sample KORA F3, and replication studies ALSPAC and EPICOR

	Discovery			Replication								
	KORA F4			KORA F3			ALSPAC			EPICOR		
	All	Males	Females	All	Males	Females	All	Males	Females	All	Males	Females
*N*	1799	877	922	500	260	240	963	499	464	536	340	196
Age range (years)	32–81	35–79	32–81	34–83	34–77	35–83	14–19	14–19	15–19	35–71	35–64	35–71
Mean age	60 (8.89)	61 (8.92)	60 (8.85)	53 (9.64)	53 (9.80)	52 (9.48)	17.10 (1.00)	17.10 (1.10)	17.10 (1.00)	52.90 (7.38)	51.45 (6.95)	55.40 (7.45)
BMI (kg/m^2^)	28.15 (4.80)	28.39 (4.27)	27.93 (5.24)	27.16 (4.54)	27.22 (3.97)	26.55 (5.07)	22.32 (3.84)	22.48 (4.08)	22.15 (3.55)	26.65 (3.87)	26.66 (3.07)	26.63 (4.96)
% Smokers	14.58	16.23	13.02	50.00	50.00	50.00	NA	NA	NA	32.83	35.29	28.57
Alcohol intake (g/days)	15.49 (20.43)	22.93 (24.17)	8.44 (12.53)	16.11 (19.59)	21.86 (21.79)	9.17 (14.10)	NA	NA	NA	17.99 (20.13)	24.08 (20.79)	7.43 (13.54)
Triglycerides (mmol/L)	1.52 (1.08)	1.72 (1.28)	1.34 (0.81)	1.88 (1.39)	2.18 (1.67)	1.56 (0.92)	0.84 (0.37)	0.83 (0.32)	0.84 (0.41)	1.72 (1.06)	1.81 (1.02)	1.57 (1.12)
Total cholesterol (mmol/L)	5.73 (1.02)	5.58 (1.00)	5.87 (1.01)	5.70 (0.99)	5.61 (1.03)	5.80 (0.93)	3.74 (0.68)	3.91 (0.69)	3.56 (0.63)	6.13 (1.19)	5.99 (1.17)	6.39 (1.19)
HDL (mmol/L)	1.46 (0.38)	1.32 (0.33)	1.59 (0.37)	1.51 (0.46)	1.33 (0.40)	1.69 (0.45)	1.25 (0.30)	1.32 (0.32)	1.18 (0.26)	1.48 (0.39)	1.38 (0.34)	1.65 (0.41)
LDL (mmol/L)	3.62 (0.91)	3.57 (0.88)	3.66 (0.93)	3.39 (0.86)	3.39 (0.84)	3.38 (0.87)	2.10 (0.60)	2.21 (0.63)	1.99 (0.55)	3.87 (1.05)	3.78 (1.02)	4.02 (1.09)
% Physical activity												
No	31.27	26.97	23.97	32.20	25.38	16.25	NA	NA	NA	21.27	15.88	30.61
Low	11.52	28.23	35.14	18.00	24.62	33.33	NA	NA	NA	43.84	41.18	48.47
Medium	31.78	12.23	10.85	28.80	18.85	17.08	NA	NA	NA	17.54	21.76	10.2
High	25.43	32.57	30.04	21.00	31.15	33.33	NA	NA	NA	17.35	21.18	10.72
% Diabetic	9.23	10.27	8.24	6.20	8.46	3.75	NA	NA	NA	1.68	1.18	2.55
% MI	3.562	5.59	1.63	1.60^a^	2.31^a^	0.83^a^	NA	NA	NA	52.98	53.23	52.55

Values given as mean (SD), otherwise indicated

^a^Myocardial infarction with hospital treatment (self-reported)

### Validation of sex-methylation associations in whole blood

**Fig. 2:**
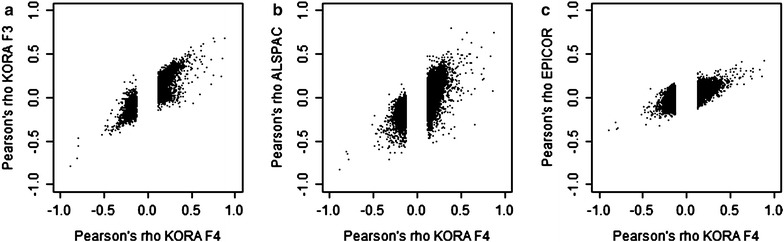
Correlations between SMAs identified in KORA F4 and the respective associations in each replication study. Each *point* corresponds to one CpG site. SMAs in the discovery sample KORA F4 are plotted against SMAs in the three replication studies, with **a** KORA F4 against KORA F3, **b** KORA F4 against ALSPAC, **c** KORA F4 against EPICOR. Note that only the CpGs that were significant in KORA F4 and subsequently taken to replication are plotted, which results in the gap in the middle of the graphs

We took the set of 11,010 SMAs forward to replication in three different cohorts coming from three different populations in three different geographic locations (Table 1, further details in the methods section). The replication results were meta-analyzed using a random-effects model, revealing 1184 CpGs (*p* < 4.89E−06). Effect sizes of revealed for these CpGs are in a range between −0.89 and 0.73 (given as theta values in Additional file 2). The histogram in Additional file 2 shows the frequency distribution of the effect sizes. Of note, the majority of the not replicated SMAs still showed consistent effect direction in the replication as in the discovery step (*p* < 1E−200, Binomial test), and SMAs were highly correlated between discovery sample and each of the three replication cohorts (correlation coefficients between KORA F4 and KORA F3, ALSPAC, EPIC, respectively, were 0.88, 0.65, 0.70), suggesting that the majority of the discovered SMAs are unlikely to be false positives (Fig. 2; Additional file 2).

### Validation in buccal epithelial tissue

Since the identification of SMAs in whole blood may be biased by cell-type composition or other tissue-specific biases, we compared the SMAs found in KORA F4 to a public dataset on DNAm measured in buccal epithelial cells from 15-year-old adolescents (*n* = 109, 60 % females) with the Illumina Infinium HumanMethylation27 BeadChip [18]. Since the buccal cells study was performed on the HumanMethylation27 BeadChip, only a subset of the sites were measured in both studies and passed the KORA quality criteria (see “Methods”): a set of 22,773 CpGs remained. Out of these 22,773 CpGs, 912 were significantly associated with sex in KORA F4, and 96 CpGs were significantly associated with sex in the buccal cells study (both after Bonferroni with *n* = 22,773, resulting significance level *p* = 2.20E−6). Despite the low rate of associations observed in the buccal cells study, we note that out of the 96 sex-associated CpGs in the buccal cells study, 16 CpGs were also associated with sex in KORA F4 (Additional file 3) (*p* value <2.4E−07, hypergeometric test). Notably, most associations in the KORA F4 were not replicated in the buccal cells study, potentially due to the limited power, with the sample size being small (*n* = 109), and due to the differences between the populations, particularly the age distribution of the subjects.

### Enrichment of SMAs in specific CpG island locations

We further tested whether there are specific regions in the genome that are enriched for SMAs by inspecting the tendency of SMAs to occur at specific locations with respect to CpG islands (CGIs). Since correlations between CpG sites can inflate the *p* value of an enrichment analysis, we randomly picked each site to participate in this analysis with probability 0.2. A set of 78,734 sites was therefore randomly chosen, out of which 231 sites are a subset of the 1184 CpGs identified in the meta-analysis. Table 2 shows a clear concentration of SMAs in CGI shores (north and south combined; Pearson Chi square test for overrepresentation, *p* value <1E−16), as opposed to a lower rate of SMAs in CGIs, shelves (north and south combined) and open sea CpG sites [19]. When excluding CGI shores from the test, there was no longer significance for any enrichment of SMAs among the remaining CGI locations (Pearson Chi square test, *p* value 0.75). These enrichment test results imply that the SMAs are overrepresented in the CGI shores.

**Table 2:** Distribution and enrichment of sex-methylation associations (SMAs) among different CpG regions

CpG region^a^	Island	Shores^b^	Shelf	Open sea	Total
No. of CpG probes	26,159	18,888	7203	26,484	78,734
Significant SMAs	51	97	19	64	231

^a^Location with respect to the CGI: shore-0 to 2 kb from CGI, shelf-2 to 4 kb from CGI; north-upstream, south-downstream [19]

^b^North and south shores combined

### Enrichment of sex-methylation associations (SMAs) in imprinted genes

In order to investigate the distribution of SMAs across the genome further, we explored the possibility that DNAm varies in imprinted genes between sexes. Imprinted genes have one silenced copy through DNAm in a parent-of-origin-specific manner; some examples of relations between autosomal DNAm patterns and the sex of the carrier of the imprinted gene are already known [20, 21]. We observed that SMAs tend to be of higher significance among CpGs at known imprinted genes compared to non-imprinted genes (Spearman correlation 0.025, *p* = 4.0E−04 considering discovery *p* values; 0.019, *p* = 8.0E−03 considering replication *p* values). Particularly, there were 3816 genes that passed the threshold for genome-wide significance in KORA F4 (where the best CpG near the gene had genome-wide significant *p* value), while out of those, 16 were imprinted genes (*p* value 0.008, hypergeometric test). Of these 16 genes, 10 also had significant SMAs in the meta-analysis; these were *GNAS*, *INPP5F*, *KCNQ1DN*, *GRB10*, *KCNQ1*, *DLGAP2*, *DLK1*, *PPP1R9A*, *MEG3*, and *PLAGL1*. This result suggests that imprinted genes tend to harbor sex-specific CpGs more often than non-imprinted genes.

### Enrichment for GO terms among the SMAs

With the aid of gene ontology (GO) terms, we analyzed the genes annotated to the sex-associated CpGs to identify common biological roles or processes in which the products of these genes might be involved. GO term-enrichment test revealed three significantly enriched GO terms among the genes annotated to the 1184 meta-analysis hits compared with the 19,170 genes annotated to the CpGs that went into our analysis in KORA F4. These GO terms were ‘negative regulation of glycolysis,’ ‘negative regulation of systemic arterial blood pressure,’ and ‘response to protozoan’ (Table 3).

**Table 3:** Enriched GO terms among the sex-associated CpGs from the meta-analysis

GO ID	GO term	*p* value^a^	Gene^b^	No. of CpG probes^c^
GO:0045820	Negative regulation of glycolysis	3.51E−07	HDAC4	393
			IER3	39
			FBP1	14
GO:0003085	Negative regulation of systemic arterial blood pressure	1.81E−06	ADRB1	8
			CALCA	40
			SOD2	7
			IER3	39
			BBS4	4
GO:0001562	Response to protozoan	2.69E−06	SLC11A1	20
			IL12B	12
			IER3	39

^a^ *p* value for enrichment, Bonferroni-corrected significance level of 3.08E−08

^b^Genes annotated to the GO term restricted to those annotated to significant SMAs in KORA F4

^c^Number of CpG probes associated with each gene, according to Illumina’s annotation file; enrichment tests were corrected for the probe numbers (see “Methods”)

### Enrichment of SMAs in sex hormone-related genes

Among the genes annotated to CpG sites with significant SMAs, we sought to explore the enrichment of genes involved in sex hormone biosynthesis, transport, receptors, genes of other hormones with sexual dimorphisms, as well as genes known to be involved in disorders of sexual development (identified by OMIM search [22]), excluding those located on X and Y chromosomes. A list of the genes considered for this analysis is given in Additional file 4. No significant enrichment among the SMAs was found for these genes (hypergeometric test, *p* value 0.41).

### Association between SMAs and expression levels

To explore the biological consequences of the 1184 significant SMAs, we tested whether there were differences in mRNA expression levels of 16,904 genes associated with both DNAm at the validated CpGs and sex in KORA F4. We considered genes 1 Mb around each SMA and identified 2 CpG-expression associations in the discovery sample from KORA F4 (*p* < 8.55E−08). The effects of methylation and sex on the mRNA expression level were negative, i.e., expression appeared to be higher both with lower methylation values and female sex. These two associations encompassed two genes: the *cytokine-inducible SH2*-*containing protein* gene (*CISH*); and the *RAB23, member RAS oncogene family* gene (*RAB23*) (Table 4).

**Table 4:** Significant associations between SMAs and mRNA expression levels in KORA F4

Probe ID expression	Probe ID methylation	est.^b^	*p* value^c^	Methylation		Sex		Dist. CpG-gene (bp)^d^	Matched gene^a^
				est.^b^	*p* value	est.^c^	*p* value		
ILMN_1738207	cg01048561	0.16	9.85E−08	−0.03	0.03	−0.82	7.25E−08	99,832	CISH
ILMN_2346997	cg16616022	0.14	3.02E−07	−0.06	0.03	−1.70	8.16E−08	100,699	RAB23

^a^Gene annotation according to expression (not to CpG sites!)

^b^Beta estimate for the model expression = *b*_0_ + *b*_1_ × sex + *b*_2_ × methylation + *e*

^c^Bonferroni-corrected significance level of 8.55E−08

^d^Genomic distance between CpG and the matched gene

## Discussion

We characterized sex differences in autosomal DNAm in a large population-based European sample and identified 11,010 sex-methylation associations (SMAs) after filtering for the highest associated CpG site per gene. Of these, 1184 were validated in a meta-analysis of additional three independent replication cohorts. The identified CpGs were enriched in CGI shores, in imprinted genes, with three GO terms, but not in sex hormone-related genes.

### Sex-specific methylation patterns

We identified marked differences in locus-specific DNA methylation between men and women throughout the autosomes with the largest proportion of effect sizes ranging between 0.5 and 1.5, but also a considerable proportion with substantial effect sizes (−0.89 to 0.73 in the meta-analysis). This is consistent with previous findings of sex-specific DNA methylation patterns. For instance, Liu et al. explored DNAm at 20,493 CpG sites in a small alcohol-abuse study (*n* = 197), identifying one gene on chromosome 4 to be significantly differentially methylated between men and women [9]. Furthermore, a recent study with main focus on the heritability and repeatability of DNAm in whole blood found 1687 CpGs associated with sex [23]. There is a significant overlap between that study and our results: 213 of the 1687 CpGs previously reported as being associated with sex are among the hits identified in the meta-analysis reported here (*p* value <1E−17, hypergeometric test). Inoshita et al. reported 292 discovery hits in peripheral blood of which we find 41 among our meta-analysis hits (25 %) and 228 among our discovery hits (2 %) [24]. As the publication does not provide a list of 98 replicated CpGs, a comparison between replicated results of their and our hits was not possible. To extend the comparison beyond blood, we compared our results with two studies with brain samples that were also analyzed with the 450-k chip. The intersection with discovery hits in the prefrontal cortex after correction for multiple testing from Xu et al. were only 2 and 3 % compared with our discovery and meta-analysis, respectively [25]. Of note, the study by Xu et al. included only 46 subjects. This fact might explain the little overlap. Spiers and co-workers published results on DNAm in fetal brain of which 14 and 18 % of their hits overlap with our discovery and meta-analysis hits, respectively [26].

Moreover, we looked at comparisons to DNAm changes associated with diabetes and with smoking as an example of environmental influence on DNAm. The incident diabetes-associated CpGs were all not significant in our data [27]. Among the 32 CpGs associated with smoking status, the majority was not significant in our results, i.e., 22 CpGs, whereas nine were significant in our KORA F4 results, two in KORA F3 and F4, one of her CpGs was excluded from our analysis [28]. Thus, none of the hits of the smoking study survived significance level in our replication or meta-analysis.

Since the four populations examined in our study were rather heterogeneous, particularly in terms of age and genetic background, the large number of replicated SMAs indicates that the majority of DNAm differences between the sexes are stable over time and independent of geographic origin.

### SMAs are found to be enriched in CpG island shores and imprinted genes

Our results on the local agglomeration of CpGs revealed an enrichment of SMAs in CGI shores. It is thought that methylation of CpGs results in different functional consequences depending on their genetic location. For example, CpGs in the gene body are thought to be related to regulation of the gene’s expression, whereas CpGs in a gene desert are thought to contribute to genomic stability [29]. With regard to CGI shores, methylation of these features seems to regulate gene expression, possibly by silencing effects [30]. However, according to Liu et al., a CpG location in genetic region is only a weak approximation to its functional consequence [31], which may explain the low number of gene expression differences linked to sex-specific DNAm in our study.

Imprinting is a phenomenon of monoallelic gene silencing through DNAm in a parent-of-origin-specific manner [32]. DNAm differences in imprinted genes have been widely reported [33–37]; some studies showed sex-specific effects of these DNAm differences [20, 21]. Looking systematically at differences of DNAm between males and females at imprinted genes, we observed an enrichment of these 10 imprinted genes among significant SMAs in the meta-analysis: *GNAS*, *INPP5F*, *KCNQ1DN*, *GRB10*, *KCNQ1*, *DLGAP2*, *DLK1*, *PPP1R9A*, *MEG3*, and *PLAGL1*. Full names of these genes and those additionally found in the discovery study are listed in Additional file 5. Although examples of sex-specific DNAm differences in imprinted genes have been reported, these differences seem to be unknown for the imprinted genes reported here.

### Three GO terms were enriched for SMAs

To achieve a more functional interpretation of the SMAs, enrichment was observed for three GO terms among the genes annotated to the CpGs in our meta-analysis: ‘negative regulation of glycolysis,’ ‘negative regulation of systemic arterial blood pressure,’ and ‘response to protozoan.’ Regarding the regulation of glycolysis, there are indications of differences between men and women in terms of glucose metabolism in the literature [38, 39]. Also, sex differences in the regulation of the arterial blood pressure are reported [40, 41]. These complementary strands of evidence suggest that sex-associated differences in DNAm may explain some of the underlying discordance in these, and possibly other traits and diseases.

### The relationship of sex hormones and DNA methylation

An analysis for enrichment of genes related to sex hormone pathways (e.g., synthesis enzymes) revealed nothing of note in our data, suggesting that sex hormone-related autosomal genes show no sex-dependent DNAm differences. Sex hormones may, however, play an important role in determining DNAm levels; steroid hormones might mediate the transcriptional effects of co-regulatory complexes that associate with epigenetic modifiers [42]. Sex hormones might also exert regulatory effects on DNAm via downregulation of DNA methyltransferases expression in certain tissues [43]. Our observations of sex-dependent DNAm differences may reflect the evidence that steroidal hormones can influence DNAm, rather than vice versa.

### Sex-associated methylation alters mRNA expression at some loci

Two genes, *CISH* and *RAB23*, showed significant DNAm-expression associations between men and women in the KORA F4 discovery sample. *CISH* and *RAB23* both exhibited lower levels of mRNA in women compared to men.

The function of RAB23 in humans is not completely unraveled yet, but it is known that this small GTPase acts as a negative regulator of the hedgehog pathway [44, 45]. Hedgehog signaling has a crucial role in response to injury, tissue stress, healing, and regeneration by helping to maintain and expand somatic stem cell populations [46]. Thus, hedgehog signaling imperatively needs to be tightly regulated. The relevance of sex-specific regulation of gene expression of this locus is unclear.

CISH is a member of the SOCS family and, together with SOCS1, 2, and 3, exhibits inhibitory functions as negative feedback to the JAK-STAT signaling pathway, triggered by cytokines like IL-2 and other stimuli [47–49]. This regulation is related to regulatory T-cell function and involved in immune polarization. Aberrations in the JAK-STAT pathway may be involved in hematopoietic disorders, autoimmune and inflammatory diseases [48], and affect susceptibility to infectious diseases [47, 49, 50].

Indeed, many infectious diseases appear to be more common in males that in females [51], but autoimmune diseases affect more females than males [52]. Thus, we hypothesize that DNAm might be a possible contributor to immunologic differences between the sexes.

Regarding the low number of genes expression of which was associated with differential DNAm in our study, one should have in mind that the consequences of changes in DNAm cover a much broader spectrum including effects in trans and genomic stability, for instance.

### Strength and limitations

Important strengths of this study are the relatively large sample size and replication in several independent studies. In addition, validation was conducted in another tissue. Furthermore, we implemented a rather stringent and conservative threshold for statistical significance so that we likely underestimated the number of SMAs reported. Both the comprehensiveness in design along with several in-depth downstream analyses strengthens our study.

Some limitations arise from the methodology of the Illumina Infinium HumanMethlation450 BeadChip which covers only 1.7 % of known CpG sites across the genome, rather than more comprehensive sequence-based approaches. However, the 450-k chip is currently the most suitable method for high-throughput measurements for large epidemiologic studies in terms of sample throughput and expenses, and the chip serves a purpose in identifying differences in DNAm at the group level, despite its widely recognized technical limitations and idiosyncrasies.

Blood is an easily accessible source of DNA in epidemiologic studies, but tissue-specificity is an important issue when analyzing DNAm. Blood may function as surrogate tissue for systemic constitutional DNAm differences, but may not necessarily mirror specific changes in other tissues [29]. We assessed tissue specificity by comparing our results to a study in buccal cells. Despite the low rate of associations observed in the buccal data, out of the 96 sex-associated CpGs in the buccal data, 16 CpGs were also associated with sex in KORA F4. However, we must underline that potential sex-related differences in white blood cell proportions might not be captured by the applied estimation method and may thus confound our analysis to some extent.

## Conclusion

We identified 1184 CpGs showing stable DNA methylation differences between men and women in four European cohorts. These sites were found to be enriched at CGI shores and at imprinted genes. Furthermore, we observed enrichment for three GO terms. From these results, we conclude that sex-dependent DNAm may be implicated in the observed sex discordance in various traits and diseases. Functional associations were demonstrated through mRNA expression analysis, which revealed two genes with significant sex- and DNAm-dependent expression differences, namely, *CISH* and *RAB23*.

Based on the substantial DNAm differences we found between men and women, we advocate that greater attention should be paid to sex differences in epigenetic studies. Further work should be conducted to establish whether the extensive catalog of sex-associated DNAm differences observed are mediating the known sex discordance seen in many traits and diseases.

## Methods

### Study populations

#### Discovery

For the identification of SMAs, we used KORA F4 as the discovery sample and meta-analyzed the results from KORA F3, ALSPAC, and EPICOR.

*KORA* (*Cooperative Health Research in the Region of Augsburg*) is a population-based research platform with subsequent follow-up studies in the fields of epidemiology, health economics, and health care research [53]. Four surveys were conducted with participants living in the city of Augsburg and 16 surrounding towns and villages. The discovery dataset comprised 1799 individuals from the KORA F4 survey (all with genotyping data available) conducted during 2006–2008. Fasting blood samples were drawn into serum gel tubes in the morning. Further details are published elsewhere [54–56]. See “Array-based DNA methylation analysis” for the laboratory analysis details.

#### Replication

As one replication sample, we used 500 subjects of the *KORA F3* (50 % smokers, age comparable to KORA F4 sample, 50 % females), originally selected for the smoking study by Zeilinger et al. [28, 56, 57].

KORA F4 and F3 are found to be completely independent with no overlap of study subjects. Also, no indication of population stratification was seen in numerous publications on the KORA studies [58]. For all studies, we obtained written consent from participants and approval from the local ethical committees.

The *Avon Longitudinal Study of Parents and Children (ALSPAC)* is a longitudinal birth cohort in the Bristol area of the UK (details in [59, 60]). A subgroup of 963 ALSPAC children samples collected in adolescence was used for replication in this study. DNA methylation data were generated on this subgroup as part of the Accessible Resource for Integrated Epigenomic Studies (ARIES) [61]. Use of the data provided has previously been approved by the ALSPAC Ethics and Law committee. Please note that the study website contains details of all the data that are available through a fully searchable data dictionary (http://www.bris.ac.uk/alspac/researchers/data-access/data-dictionary/).

A third replication sample was from a nested case–control study from the Italian *EPICOR2* study [part of the European Prospective Investigation into Cancer and Nutrition (EPIC) Study]. The study sample includes 292 myocardial infarction (MI) cases and 292 matched controls that were healthy at recruitment, but diagnosed for nonfatal MI during the follow-up. Blood samples were collected at the time of enrollment. Further details are published in Fiorito et al. [62].

### Array-based DNA methylation analysis

Genome-wide DNAm patterns of the 1814 KORA F4 samples (1 µg DNA from whole blood) were assessed using the Infinium HumanMethylation450 BeadChip Array (Illumina) as described elsewhere [28]. Also in the replication studies, DNAm was measured with the 450-k chip.

The percentage of methylation of a given cytosine is reported as a beta-value, which is a continuous variable between 0 and 1, corresponding to the ratio of the methylated signal over the sum of the methylated and unmethylated signals.

Technical validation of the method has been reported elsewhere [63].

### Data preprocessing and quality assurance

Raw methylation data were obtained from GenomeStudio software (Illumina, version 2011.1) methylation module (version 1.9.0) and preprocessed as proposed by Touleimat and Tost [64] with default option and an in-house updated list of CpGs with SNPs in the probe-binding regions, followed by beta-mixture quantile normalization (BMIQ) as a normalization step to correct for the InfI/InfII distribution shift of the beta-values ([65], using R-package wateRmelon, version 1.0.3 [66]). After the quality control, 391,885 CpGs for 1799 samples were eligible to enter the analysis. Details on data normalization can be found in Wahl et al. [67].

For the ALSPAC study, ARIES DNA methylation data version 1, released in October 2013, was used. During quality control, samples were excluded if they had detection *p* value ≥0.01 at more than 20 % probes, or unexpected mismatches with SNP genotype data or sex information. Finally, for normalization, the Touleimat and Tost method implemented in R-package *wateRmelon*, version 1.0.3 was applied with default parameters.

Similar procedures as in KORA were done in the EPICOR study, resulting in another 788 CpGs being excluded from the given KORA F4 hits.

### Statistical analysis

We defined the sex-methylation association SMA at each CpG site to be the Pearson’s correlation between sex and the methylation beta-value. Prior to association testing, methylation measurements were adjusted across the study sample by taking the residuals of the linear regression with the following covariates: Age, BMI, smoking behavior, alcohol intake, triglycerides, total leucocytes, plate, HDL, LDL, physical activity, diabetes, myocardial infarction, and estimated white blood cell proportions. Specifically, the proportions of neutrophils, CD4^+^ T, CD8^+^ T, B cells, monocytes, and granulocytes were estimated according to Houseman et al. [68]. Experimental 96-well plates were represented by dummy variables. The Manhattan plot was drawn with an adapted version of the drawing function in the qqman package for R [69].

### Allosomal cross-hybridization

Previous studies reported a subset of the 450k probes to be cross-reactive in silico [70, 71]. Cross-hybridization with the sex chromosomes leads to false discoveries as the measured methylation value is composed of a mixture of values from the target location and from the sex-chromosome, which obviously differs between sexes. Price et al. showed that probes with high homology to the sex chromosomes have a significant enrichment of sex-methylation associations [71].

We repeated the computational analysis of Price et al. in order to validate that the 450k probes have no correlation between allosomal homology and sex associations in our dataset. For each autosomal probe, we defined the cross-hybridization value to be equal to the number of matches between each probe and its best alignment to the allosomes. The cross-hybridization value equals the number of matching bases between each probe sequence and its best alignment to chromosomes X and Y. We observed that the highly significant SMAs tend to have high cross-hybridization values (see Additional file 6). We therefore removed all CpGs from the analysis with cross-hybridization values larger than a threshold of 40 (the point at which enrichment of SMAs starts). After the removal of these CpGs, the correlation between the SMA values and the cross-hybridization values was not statistically significant (*p* values were calculated empirically using 3000 permutations). This process resulted in the removal of 12,260 probes, of which 568 had significant sex associations (4.63 %). To be conservative, we additionally removed all nonspecific probes suggested by price; 391,885 CpGs were finally considered as safe probes.

### Replication and meta-analysis

For replication of the significant CpGs from the discovery step, three studies were included: KORA F3, ALSPAC, and EPICOR. Association analysis was performed as described for the discovery sample. Covariates were included as follows: In KORA F3, all of the discovery covariates were included. In ALSPAC, age, BMI, total cholesterol, HDL-C, LDL-C, tissue contents estimated by the Houseman algorithm [68], and the bisulfite conversion batch (BCD plate) were included. In EPICOR, the discovery covariates were complemented with the center of recruitment (categorical: Torino, Varese, Napoli, Ragusa). Results from the three replication studies were meta-analyzed using the R function *metacor* (with default settings, i.e., Fisher’s z-transformation of Pearson’s rho values, random effects model). Significance level in the meta-analysis was Bonferroni-corrected for 10,222 tests corresponding to the number of CpGs analyzed in all replication cohorts (i.e., 4.89E−06).

### Validation in monocellular tissue (buccal epithelium cells)

From the GEO database, we chose the largest publicly available dataset from a population-based study with even sex distribution and monocellular tissue as study sample [18]. In that study, the Illumina Infinium HumanMethylation27 BeadChip v1.2 was applied on buccal epithelium cells, and the data is publicly accessible at NCBI GEO database [72], Accession GSE25892.

The following preprocessing steps were already completed: Extraction of raw intensities using GenomeStudio v2010.1, background normalization by subtracting averaged negative probe intensity from signal A and B, quantile normalization, obtaining beta-values, and checking CpG site-wise call rate (CpGs removed if >50 % of data points had bad quality). According to our data preprocessing in KORA, we additionally set beta-values to missing where respective detection *p* values were 0.01 or below, excluded samples with call rates of ≤80 % (there were none). BMIQ was not necessary since it is designed to correct the InfI–InfII distribution shift, which is not an issue when 27k is used, where all CpGs are of InfI design.

We obtained a subset of 22,773 CpG sites, which were measured in both experiments, and passed the above quality-control steps. We tested for SMAs in the buccal dataset with a linear model as no other covariates were available. We used a conservative Bonferroni correction for 27k hypotheses throughout this analysis.

### Test for enrichment of SMAs in CpG locations

CGI location information was taken from UCSC genome browser CGI specification which was available from Illumina’s probe description file. Since test for enrichment assumes independency (both the hypergeometric test and the Spearman correlation), and since CpG sites are locally correlated, we preprocessed the data by analyzing a random subset of the sites which was obtained by picking each site with probability 0.2. Applying this filter resulted in 78,734 CpG sites, 231 of these had significant SMA after replication.

### Enrichment of SMAs in imprinted genes

We tested a known set of imprinted genes for enrichment of significant SMA CpGs. For that purpose, we downloaded a list of 59 genes from ‘Geneimprint’ by Dec 2013 [73] and selected only those with status ‘imprinted’ in Homo sapiens [74, 75]. Of these genes, 48 had CpG sites that passed quality control. For each gene as to Illumina’s Infinium 450k annotation, including these 48, we picked the most significant CpG sites in our analysis, resulting in 19,170 sites. We considered the vector of *p* values for these sites, and then considered another binary vector of length 19,170, where an entry is 1 if the corresponding gene is an imprinted gene. Spearman correlations were calculated for the pairs of vectors.

### GO-enrichment analysis

An enrichment analysis for gene ontology (GO) terms was performed with all CpGs analyzed in KORA F4, resulting in 19,170 annotated genes (according to Illumina’s annotation file). GO is an ontology of defined terms representing gene product properties [76]. According to Geeleher et al. [77], we applied the R-package *GOseq* [78], originally developed for expression analysis, to determine different probabilities of detecting a gene due to different CpG probe number per gene. Therefore, we defined those CpGs as differentially methylated which were replicated in the meta-analysis, looked at the annotated genes, and used the number of probes per gene as variable for weighting the results. Results were Bonferroni-corrected for 16,233 tests according to the number of genes that were processed by the *GOseq* function.

### Enrichment in sex hormone-related genes

We created a list of genes involved in sex hormone biosynthesis, transport, receptors, genes of other hormones with sexual dimorphisms, as well as genes known to be involved in disorders of sexual disorders (identified by an OMIM search [22]), excluding those located on X and Y chromosomes (Additional file 4). We then compared those to our meta-analysis hits with a hypergeometric test.

### mRNA expression analysis

Gene expression data were available for 731 KORA F4 subjects and were analyzed using the Illumina Human HT-12 v3 Expression BeadChip. The procedures are described elsewhere [79]. For all CpGs surpassing the threshold for statistical significance in the meta-analysis, mRNA expression levels were checked as expression of genes in 1 Mb distance to each CpG site as linear combination of sex effect and DNAm effect with the same co-factors as in the main analysis. The significance level of 8.55E−08 was calculated as 0.05 divided by the number of combinations between CpGs and genes, which was 585,031.

### Software

Analyses were performed using Matlab and the Statistics Toolbox, Release 2013b, The MathWorks, Inc., Natick, Massachusetts, United States. Preprocessing and quality control steps as well as replications, meta-analysis, expression analysis, and GO and sex hormone-related gene-enrichment analyses were done using R, versions 2.15.3 3.0.1, and for the Manhattan plot version 3.1.3 [80].

## Additional files

10.1186/s13072-015-0035-3 Flow-chart of the studies and analyses. The figure provides an overview of the analyzed studies and the conducted analyses with corresponding numbers.
10.1186/s13072-015-0035-3 Results from discovery (KORA F4) and replication analyses (KORA F3, ALSPAC, EPICOR). The table includes respective Pearson’s rho and p-values for 391,885 CpGs in KORA, 11,010 CpGs in ALSPAC, and 10,222 CpGs in EPICOR as well as theta values and p-values for the meta-analysis. The file additionally contains a figure showing the frequency distribution of the effect sizes from the meta-analysis. 
10.1186/s13072-015-0035-3 Sex-associated CpGs in KORA F4 and the buccal cells study. The table includes CpGs significantly associated with sex in the KORA F4 and in the buccal cells study with chromosome number and position, CGI region, and annotated gene for each CpG, beta estimates and p-values per study.
10.1186/s13072-015-0035-3 Sex-related genes considered for enrichment analysis. Given are the official gene symbol and the corresponding chromosomes. X and Y are marked in red since they were excluded from the enrichment analysis.
10.1186/s13072-015-0035-3 Imprinted genes with significant SMAs enrichment.
10.1186/s13072-015-0035-3 Enrichment of SMAs for probes with high Cross-Hybridization value (CHV). CHV is the number of matching bases to XY chromosomes. CHV values greater than 46 tend to be enriched for SMAs (green line). To be conservative, we removed all SMAs, represented as correlation coefficients on the y-axis, with CHV > 40 (red line).

## Abbreviations

CGI: CpG island
DNAm: DNA methylation
GO: gene ontology
EWAS: epigenome-wide association study
SMA: sex methylation association
