# Supplementary figures and images for: Characterization of whole-genome autosomal differences of DNA methylation between men and women
Source: Epigenetics Chromatin. 2015 Oct 19;8:43. doi: 10.1186/s13072-015-0035-3 (PMC4615866; doi:10.1186/s13072-015-0035-3)

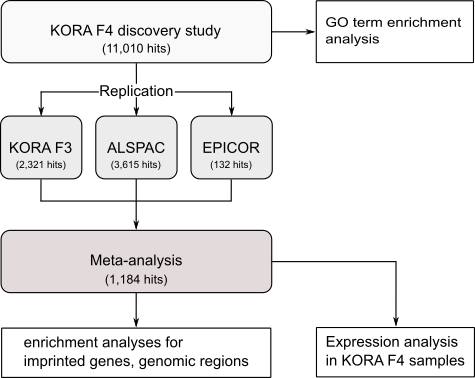

Supplement: Supplementary file 1 — 10.1186/s13072-015-0035-3 Flow-chart of the studies and analyses. The figure provides an overview of the analyzed studies and the conducted analyses with corresponding numbers. [file 13072_2015_35_MOESM1_ESM.png]

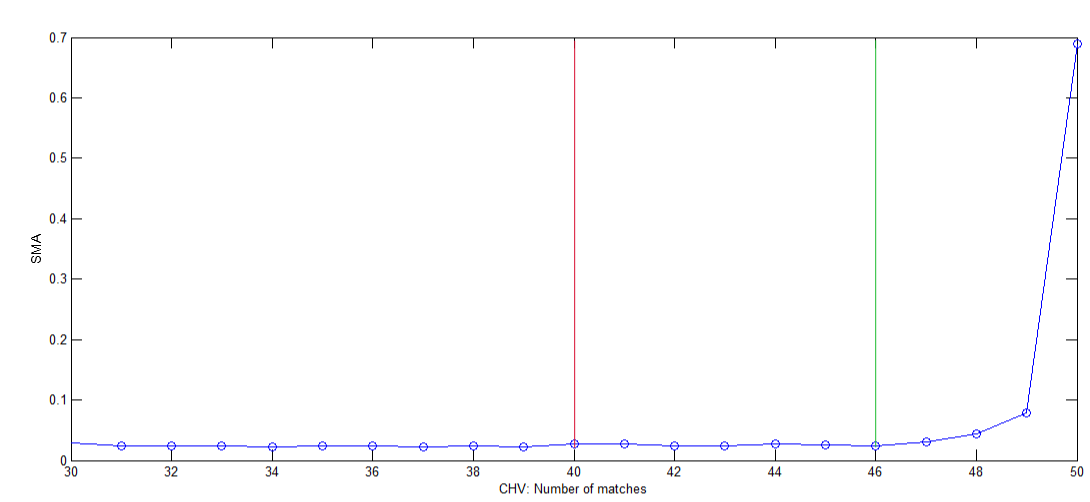

Supplement: Supplementary file 6 — 10.1186/s13072-015-0035-3 Enrichment of SMAs for probes with high Cross-Hybridization value (CHV). CHV is the number of matching bases to XY chromosomes. CHV values greater than 46 tend to be enriched for SMAs (green line). To be conservative, we removed all SMAs, represented as correlation coefficients on the y-axis, with CHV > 40 (red line). [file 13072_2015_35_MOESM6_ESM.png]
